# Multi-Omics Analysis Uncovers the Mechanism for Enhanced Organic Acid Accumulation in Peach (*Prunus persica* L.) Fruit from High-Altitude Areas

**DOI:** 10.3390/plants13223171

**Published:** 2024-11-12

**Authors:** Haiyan Song, Ke Zhao, Xiaoan Wang, Guoliang Jiang, Jing Li, Chengyong He, Lingli Wang, Shuxia Sun, Meiyan Tu, Qiang Wang, Ronggao Gong, Dong Chen

**Affiliations:** 1 Institute of Horticulture, Sichuan Academy of Agricultural Sciences, Chengdu 610066, China; lovesong@scsaas.cn (H.S.); zhaoke0607@163.com (K.Z.); wangxa0515@163.com (X.W.); jiangguoliang@scsaas.cn (G.J.); lijing@scsaas.cn (J.L.); hchyong2019@163.com (C.H.); abl13272755@126.com (L.W.); sunshuxia@scsaas.cn (S.S.); tumeiyan@scsaas.cn (M.T.); 2 Key Laboratory of Horticultural Crop Biology and Germplasm Creation in Southwest China, Ministry of Agriculture and Rural Affairs, Chengdu 610066, China; 3 College of Horticulture, Sichuan Agricultural University, Chengdu 6111130, China; rggong@sicau.edu.cn; 4 Chengdu Agricultural Technology Extension Station, Chengdu 610095, China; 13980625796@163.com

**Keywords:** peach, high altitude, organic acid, WGCNA

## Abstract

The early-ripening peach industry has undergone rapid development in the Panxi region of the Sichuan Basin in recent years. However, after the introduction of some new peach varieties to the high-altitude peach-producing areas in Panxi, the titratable acid content in peach fruit has significantly increased. This study compared the fruit quality indicators of early-ripening peach varieties cultivated in Xide County (a high-altitude peach-producing area) and Longquanyi District (a low-altitude peach-producing area) in Sichuan Province and analyzed the differences in organic acid metabolism by combining primary metabolomic and transcriptomic approaches. The results showed that the ‘Zhongtaohongyu’ fruit from the high-altitude peach-producing area had a much higher accumulation of malic acid and, accordingly, a significantly higher organic acid content than the other samples. The lower annual average temperature and stronger ultraviolet radiation in high-altitude peach-producing areas may lead to the increased expression of genes (*PpNAD-ME1*, *PpNADP-ME3,* and *PpPEPC1*) in the organic acid synthesis pathway and the decreased expression of genes (*PpACO2*, *PpNAD-MDH2/3/4/5*, and *PpPEPCK2*) in the organic acid degradation pathway in peach fruit, ultimately resulting in the accumulation of more organic acids. Among them, the downregulation of the key genes *PpNAD-MDH3/4/5* involved in malic acid metabolism may be the main reason for the higher malic acid accumulation in peach fruit from high-altitude peach-producing areas. Overall, this study elucidates the mechanism by which environmental factors enhance the accumulation of organic acids in peach fruit from high-altitude peach-producing areas from a multi-omics perspective, as well as providing a theoretical basis for screening key genes involved in organic acid metabolism in peach fruit.

## 1. Introduction

Peach originated in western China and has a cultivation history of over 5000 years. In recent years, peach cultivation areas have been gradually expanded to many low-latitude and high-altitude areas in southwest China, such as the Daliangshan area in the Sichuan Basin and the Yunnan–Guizhou Plateau [1,2,3]. However, with the increase in altitude, some challenges become apparent during the growth of different peach varieties, including an increase in titratable acid content in fruit [4], fruit surface burn caused by intense ultraviolet radiation [2], and a reduction in the fruit setting rate due to low temperatures during the flowering period [5]. Amongst these challenges, the underlying cause for the increase in organic acid content in peach fruit cultivated in high-altitude regions remains elusive.

Organic acids play a pivotal role in influencing the flavor quality of peach fruit. Malic acid is the primary organic acid component in peach fruit, constituting over 45% of the total organic acid content [6,7]. In addition, there are also a small number of peach germplasm resources whose fruits are predominantly composed of quinic acid and citric acid [8].

At the initial stage of fruit development, malic acid is synthesized within the cytoplasm and subsequently accumulated in vacuoles. At the late stage of fruit development, malic acid is released from vacuoles and degraded in the cytoplasm [9,10]. In this process, phosphoenolpyruvate (PEP) generated via the glycolytic pathway undergoes carboxylation by phosphoenolpyruvate carboxylase (PEPC) to yield oxaloacetic acid (OAA), which is subsequently converted into malic acid under the action of NAD-malate dehydrogenase (NAD-MDH) [11,12]. Therefore, PEP and NAD-MDH are generally considered as key enzymes in malic acid synthesis in fruit.

The accumulation of organic acids in peach fruit is mainly controlled by the D locus on chromosome 5 [13,14]. A gene encoding a vacuolar membrane glucose transporter (*PpTST1*, Prupe.5G006300) that regulates organic acid accumulation in peach fruit was identified through a genome-wide association analysis of resequencing data and total titratable acid content of 227 peach germplasm resources. A nonsynonymous G/T mutation at the end of the third exon of *PpTST1* resulted in two genotypes, *PpTST1^Gln^* and *PpTST1^His^*, respectively. The overexpression of *PpTST1^His^* reduced the accumulation of total organic acids and malic acid in peach flesh, while that of *PpTST1^Gln^* showed no such effect [15]. Another study of sour and non-sour peach varieties showed that a gene encoding a putative small protein, which was designated as *PpRPH* and located in the D locus, may be a key regulatory gene for the acidity of peach fruits [16]. In addition, the transcription factors *PpWRKY50* and *PpMYB62* and the cation/hydrogen ion exchanger gene *PpCHX18* are also considered candidate genes regulating malic acid content in peach fruit [17]. A recent study demonstrated that the transient overexpression of *PpALMT1*, a gene encoding an aluminum-activated malate transporter, can also promote the accumulation of malic acid in peach fruit [18]. In general, the organic acid content in peach fruit is a quantitative trait regulated by multiple genes.

The external environment may also affect the content and composition of organic acids in fruit, including temperature, water, light, mineral nutrition, ultraviolet rays, and soil salt stress [10,19]. Among these factors, temperature is considered the most critical factor affecting the accumulation and metabolism of titratable acid in fruit [20]. Low temperatures can preserve citric and malic acid levels by suppressing aconitate hydratase (ACO) and inducing malate dehydrogenase (MDH), thereby delaying flavor degradation [21]. On the contrary, an increase in ambient temperature during fruit development or storage leads to a decrease in malic acid content in peach fruit [22]. A recent study showed that more organic acids, such as quinic acid, accumulate in the peach fruits grown at high altitudes in the Tibetan region than in those grown at low altitudes, and these acids are considered potentially crucial metabolites for the adaptation of the Tibetan peach to high-altitude conditions [23]. The above studies indicate that temperature and ultraviolet radiation may affect the accumulation of organic acids in peach fruit.

Here, we compared the organic acid metabolism profiles and transcriptome profiles of peach fruits grown at different altitudes in the Sichuan Basin. Multi-omics joint analysis revealed that the up-regulation of genes involved in the malic acid synthesis pathway, along with the down-regulation of genes associated with the malic acid decomposition pathway, may be the reasons for the increased organic acid content in peach fruits from high-altitude peach-producing areas. Furthermore, a weighted gene co-expression network analysis (WGCNA) was employed to explore the potential regulatory network involved in organic acid metabolism in peach fruit.

## 2. Materials and Methods

### 2.1. Plant Materials and Growth Conditions

Two peach (*Prunus persica* L.) varieties, ‘Zhongtaojinkui’ and ‘Zhongtaohongyu’, were used as the test materials. The sampling sites were situated in a low-altitude peach-producing area (Longquanyi District, Sichuan Province, at 104°17′01″ E, 30°31′16″ N, with an altitude of 560 m, annual sunshine hours of 1033 h, an annual temperature of 16.5 °C, and an annual rainfall of 896 mm) and a high-altitude peach-producing area (Xide County, Sichuan Province, at 102°16′4″ E, 28°5′42″ N, with an altitude of 1779 m, annual sunshine hours of 2016 h, an annual temperature of 14 °C, and an annual rainfall of 1034 mm). From June to July in 2024, the fruit samples were collected at full maturity, with each material comprising ten fruit. The samples included ‘Zhongtaojinkui’ from Xide (XD-JK) and Longquanyi (LQ-JK) and ‘Zhongtaohongyu’ from Xide (XD-HY) and Longquanyi (LQ-HY). After imaging, the fruit vertical diameter, horizontal diameter, and firmness were measured using a digital vernier caliper (DL91150, Deli group Co., Ltd., Ningbo, Zhejiang, China) and a digital fruit hardness tester (GY-4, TOP Cloud-agri, Hangzhou, Zhejiang, China), respectively. Pericarps and seeds were then removed and pooled. The flesh was cut into small pieces, rapidly frozen in liquid nitrogen, and stored at −80 °C until analysis. Three biological replicates were prepared for the samples.

### 2.2. Determination of Main Indicators for Fruit Intrinsic Quality

The anthrone–sulfuric acid method was employed to detect the content of soluble sugar in the flesh. Briefly, a series of glucose standard solutions of varying concentrations was prepared, and 1 mL glucose standard solutions, 0.5 mL anthrone reagent (2 mg/mL), and 4.5 mL concentrated sulfuric acid were added to each test tube. Using the distilled water as the control, after fully shaking, the test tubes were placed into boiling water for 10 min, and then removed and allowed to cool naturally to room temperature. Subsequently, a spectrophotometer (UV-1700, Macylab, Shanghai, China) was used to measure the absorbance value at a wavelength of 620 nm and a standard curve was drawn. Next, an extraction solution of peach flesh was prepared at a concentration of 10 mg/mL, the absorbance value was measured according to the aforementioned method, and the soluble sugar content was calculated according to the standard curve. The content of titratable acid was measured using TC2303 kit (Leagene, Beijing, China) according to the manufacturer’s instructions. All experiments were performed with three biological replicates.

### 2.3. Metabolome Profiling of Primary Metabolites

The vacuum freeze-drying method was used to prepare plant samples for primary metabolite analysis. Briefly, the biological samples were placed in a lyophilizer (Scientz-100F, Scientz, Ningbo, Zhejiang, China). Following freeze-drying, the samples were ground into powder using a grinder (MM 400, Retsch, Shanghai, China) at 30 Hz for 1.5 min. Next, 50 mg of the sample powder was weighed using an electronic balance (MS105DΜ, Mettler Toledo, Shanghai, China) and 1200 μL of −20 °C pre-cooled 70% methanolic aqueous internal standard extract (added at a rate of 1200 μL extractant per 50 mg sample, with a maximum of 50 mg internal standard added) was added. The mixture was vortexed for 30 s once every 30 min, for a total of six vortexing sessions. After centrifugation at a rotation speed of 12,000 rpm for 3 min (5417R, Eppendorf, Hamburg, Germany), the supernatant was aspirated, and the sample was filtered through a microporous membrane (SCAA-104, 0.22 μm pore size; ANPEL, Shanghai, China). The sample extracts were analyzed using an UPLC-ESI-MS/MS system (UExionLC™ AD, SCIEX, Shanghai, China) and tandem mass spectrometry system (https://sciex.com.cn/, accessed on 25 July 2024). A qualitative analysis of the mass spectrum data was performed using the Metware Database (https://cloud.metware.cn, accessed on 28 July 2024). Subsequently, quantitative analysis was completed using the Multiple Reaction Monitoring (MRM) method, following the approach of Ding et al. [24].

### 2.4. Screening and Enrichment Analysis of Differentially Accumulated Metabolites

Unsupervised principal component analysis (PCA) was performed by statistics function prcomp within R (www.r-project.org, accessed on 15 August 2024). The data were unit-variance-scaled before the unsupervised PCA, as described by Shu et al. [25]. Differentially accumulated metabolites (DAMs) were identified by filtering with a *p*-value < 0.05 and a fold change ≥1.20 or ≤0.83. The identified metabolites were annotated by querying the Kyoto Encyclopedia Genes and Genomes (KEGG) compound database (http://www.kegg.jp/kegg/compound/, accessed on 15 August 2024), and the annotated metabolites were then mapped to the KEGG pathway database (http://www.kegg.jp/kegg/pathway.html, accessed on 15 August 2024).

### 2.5. Transcriptome Profiling

The total RNA was extracted for three biological replicates from the flesh of materials with a TRIzol reagent. Subsequently, the total RNA was identified and quantified using a Qubit fluorescence quantifier (Qubit 4.0, Thermo Fisher Scientific, Waltham, MA, USA) and a high-throughput biofragment analyzer (Qsep400, Bioptic, Shanghai, China). Then, sequencing was conducted using the Illumina Hiseq4000 platform (Illumina, San Diego, CA, USA), and the data were filtered to obtain clean data, which were then compared with the reference genome of peach (https://www.rosaceae.org/species/prunus_persica/genome_v2.0.a1, accessed on 29 July 2024) for sequence alignment and annotation, following the method of Song et al. [26]. mRNA expression levels were determined by calculating the number of fragments per kilobase of transcript per million fragments mapped (FPKM).

### 2.6. Analysis of Differentially Expressed Genes

The differentially expressed genes (DEGs) of four peach samples were filtered out with∣Log_2_ fold change∣ ≥ 1 and *p*-value < 0.05. Finally, 3337 DEGs were obtained from 4 datasets and Gene Ontology (GO) enrichment analysis was performed using the topGO R package (http://www.bioconductor.org/packages/release/bioc/html/topGO.html, accessed on 15 August 2024). Then, the 1530 DEGs from ‘Zhongtaojinkui’ and 1102 DEGs from ‘Zhongtaohongyu’ were annotated using the Kyoto Encyclopedia Genes and Genomes (KEGG) database (http://www.kegg.jp/kegg, accessed on 15 August 2024). The annotated metabolites were then mapped to the KEGG pathway database.

### 2.7. Analysis of Expression Patterns of Key Genes Involved in the Organic Acid Metabolic Pathway

The RNA-seq data for four peach samples were obtained from the NGDC repository (https://ngdc.cncb.ac.cn/gsa, accessed on 20 September 2024) with the accession number of CRA019041. A local database was constructed using the published peach genome of Chinese cling [27]. Key genes involved in the organic acid metabolism pathway in peach were identified using BlastP and the NCBI Conserved Domain Database (CDD) following the method of Song et al. [28]. Pathway maps illustrating the key metabolites and genes related to organic acid metabolism were generated using TBtools [29] and Adobe Illustrator 2021.

### 2.8. RNA Extraction and Real-Time Quantitative Polymerase Chain Reaction Analysis

The total RNA was extracted from the peach flesh following the method described previously in transcriptome profiling. The real-time quantitative polymerase chain reaction (RT-qPCR) procedure followed the SYBR^®^ Premix Ex Taq manual (Takara, Dalian, China). The relative gene expression levels were calculated using the 2^−ΔΔCt^ method. The primers used in the RT-qPCRs are listed in Table S1.

### 2.9. Weighted Gene Co-Expression Network Analysis Between Transcriptome and Three Organic Acid Traits

The Plant Transcription Factor Database (http://planttfdb.gao-lab.org/, accessed on 20 September 2024) and iTAK (http://itak.feilab.net/cgi-bin/itak/index.cgi, accessed on 20 September 2024) were used to annotate all transcription factors (TFs) identified in the peach genome of Chinese cling [27]. By using the cloud platform of the Metaware Company (https://cloud.metware.cn, accessed on 20 September 2024), the Pearson correlation coefficient was calculated for the transcriptome, metabolome, and three organic acid traits. Then, based on the correlation with total titratable acid content, the two modules with the highest positive and negative correlations were screened out, and from these modules, the ten transcription factors with the highest average FPKM values were selected. The correlation between the transcription factors, structural genes, and three organic acid traits was calculated, and Cytoscape v3.10.0 [30] was used to generate the network diagram.

## 3. Results

### 3.1. Comparison of Fruit External Appearance and Internal Quality Indicators

**Figure 1:**
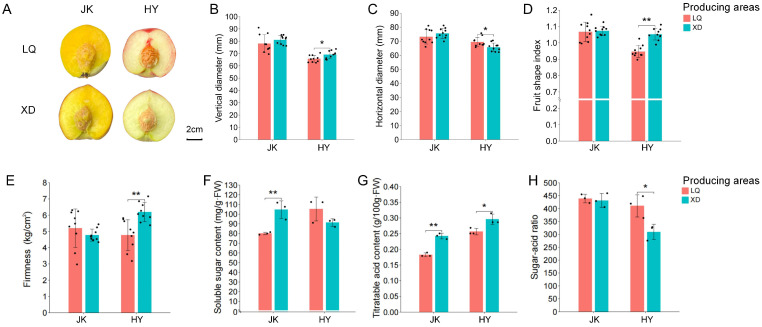
External appearance and internal quality indicators of the fruits of ‘Zhongtaojinkui’ and ‘Zhongtaohongyu’ from two peach-producing areas at different altitudes. (**A**) Comparison of the longitudinal section of the fruit. (**B–H**) Comparison of the fruit’s vertical diameter (**B**), horizontal diameter (**C**), fruit shape index (**D**), firmness (**E**), soluble sugar content (**F**), titratable acid content (**G**), and sugar-acid ratio (**H**). LQ stands for ‘Longquanyi’, XD stands for ‘Xide’, JK stands for ‘Zhongtaojinkui’, and ‘HY’ stands for ‘Zhongtaohongyu’. One or two asterisks indicate statistical significance according to Student’s *t*-test at the 0.05 or 0.01 level, respectively.

The external appearance and internal quality indicators of peach fruit from two peach-producing areas located within the Sichuan Basin were first compared. As shown in Figure 1, compared with LQ-HY, XD-HY exhibited a significantly larger vertical diameter and smaller horizontal diameter, resulting in an extremely significantly higher fruit shape index (Figure 1B–D). Moreover, XD-HY had significantly higher fruit firmness than LQ-HY (Figure 1E). In contrast, XD-JK and LQ-JK exhibited no significant difference in the above three indicators.

Moreover, XD-JK had significantly higher soluble sugar and titratable acid contents than LQ-JK. No significant difference was observed in the soluble sugar content between XD-HY and LQ-HY, but XD-HY had a significantly higher titratable acid content than LQ-HY, leading to a significantly lower sugar–acid ratio (Figure 1F–H). Thus, altitude had a pronounced impact on both the external appearance and internal quality attributes of peach fruit, particularly in terms of titratable acid content.

### 3.2. Analysis of Primary Metabolites

**Figure 2:**
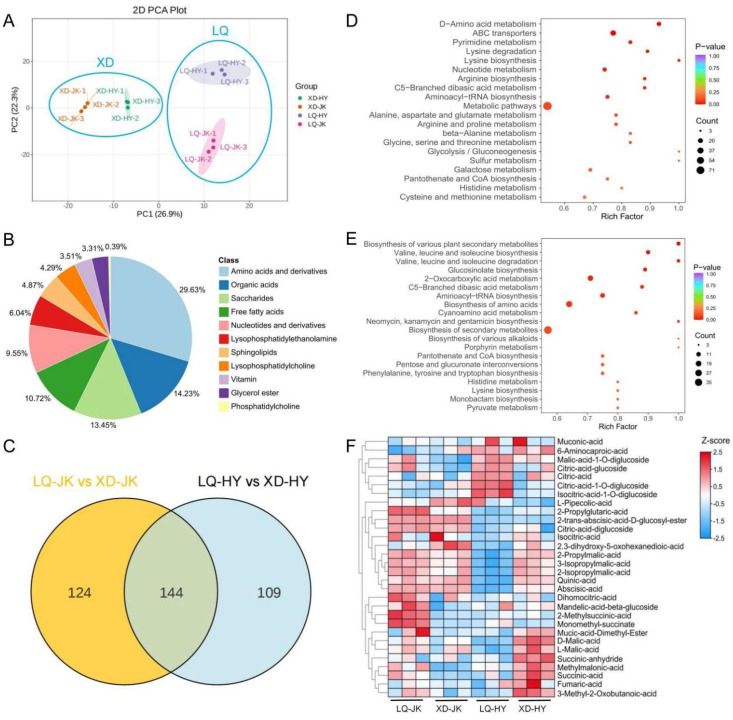
Primary metabolite profiles of the fruits of ‘Zhongtaojinkui’ and ‘Zhongtaohongyu’ from two peach-producing areas at different altitudes. (**A**) Result of PCA for four metabolome datasets. (**B**) Classification and proportion of metabolites detected in all samples. (**C**) Differentially accumulated metabolites of XD-JK and XD-HY, relative to LQ-JK and LQ-HY, respectively. (**D**,**E**) KEGG pathway enrichment analysis of differentially accumulated metabolites in ‘Zhongtaojinkui’ and ‘Zhongtaohongyu’ from two peach-producing areas at different altitudes. (**F**) Profiles of organic acids in the fruits of ‘Zhongtaojinkui’ and ‘Zhongtaohongyu’ from two peach-producing areas at different altitudes.

To elucidate the differences in primary metabolites in the two peach varieties grown in peach-producing areas at different altitudes, a metabolome analysis of primary metabolites was conducted. PCA was performed on the metabolome samples of XD-JK, XD-HY, LQ-JK, and LQ-HY (as shown in Figure 2A). The samples were distinctly separated into four groups based on their metabolic profiles. Specifically, the samples from two peach-producing areas were clearly clustered into two distinct groups, and XD-JK and XD-HY were also clearly separated from each other along PC1, which explained 26.9% of the total variance. Furthermore, LQ-JK and LQ-HY were distinctly separated into two groups along PC2, which accounted for 22.3% of the total variance. These results suggested that altitude has a considerable impact on the accumulation of primary metabolites in the flesh of peach fruit.

The metabolomic analysis identified a total of 513 metabolites, including 152 amino acids and derivatives, 152 lipids, 73 organic acids, 49 nucleotides and derivatives, and 87 other unclassified metabolites (Table S2). Notably, under the secondary classification of primary metabolites, the top two metabolites were amino acids and derivatives (152) and organic acids (73), accounting for 29.63% and 14.23% of all of the annotated metabolites, respectively (Figure 2B).

### 3.3. Screening and Analysis of Differentially Accumulated Metabolites

To investigate the potential influence of altitude on the accumulation of DAMs in the flesh of peach, the metabolomic data of primary metabolites in four samples were analyzed. Relative to LQ-JK and LQ-HY, XD-JK and XD-HY had 144 common DAMs and 124 and 109 unique DAMs, respectively (Figure 2C). The KEGG pathway enrichment analysis of ‘Zhongtaojinkui’ demonstrated that these metabolites were predominantly enriched in 20 pathways, such as ‘D-Amino acid metabolism’, ‘alanine, aspartate and glutamate metabolism’, and ‘Glycolysis/Gluconeogenesis’ (Figure 2D). Moreover, the ‘Pentose and glucuronate interconversions’, ‘2-Oxocarboxylic acid metabolism’, and ‘Pyruvate metabolism’ pathways, among others, were enriched in the flesh of ‘Zhongtaohongyu’ (Figure 2E). These results indicated that the variation in the titratable acid content of peach fruit from two different altitudes might be attributed to differences in the content of metabolites involved in organic acid metabolic pathways.

To observe the differences in organic acid accumulation in four samples from different peach-producing areas, a heat map was generated using metabolomics data to analyze the differentially accumulated organic acids (Figure 2F). The results showed that altitude had a significant effect on the composition and content of organic acids in peach fruit. The primary organic acids were 2-propylglutaric acid, 2-methylsuccinic acid, and monomethyl succinate in LQ-JK, and were L-pipecolic acid, isocitric acid, and 2,3-dihydroxy-5-oxohexanedioic acid in the XD-JK samples. The top five organic acids were malic acid-1-O-diglucoside, citric acid–glucoside, citric acid, citric acid–1-O-diglucoside, and isocitric acid–1-O-diglucoside in LQ-HY, and D-malic acid, L-malic acid, succinic anhydride, methylmalonic acid, and succinic acid in XD-HY. Evidently, XD-HY exhibited a significant accumulation of malic acid, which contributed to its higher titratable acid content.

### 3.4. Transcriptome Analysis

**Figure 3:**
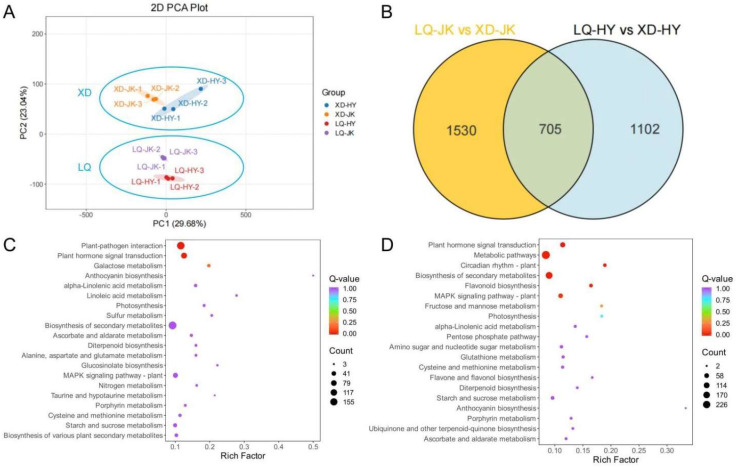
The transcriptome profiles of the fruits of ‘Zhongtaojinkui’ and ‘Zhongtaohongyu’ from two peach-producing areas at different altitudes. (**A**) The result of PCA for four transcriptome datasets. (**B**) The differentially expressed genes of XD-JK and XD-HY, relative to LQ-JK and LQ-HY, respectively. (**C**,**D**) KEGG pathway enrichment analysis of differentially expressed genes in ‘Zhongtaojinkui’ and ‘Zhongtaohongyu’ from two peach-producing areas at different altitudes.

RNA-seq was utilized to investigate the transcriptome data of all four tested samples. PCA was conducted on the transcriptome profiles and the results are shown in Figure 3A. Based on their transcriptome data, the samples were distinctly clustered into four groups. The four samples were not separated along PC1, which explained 29.68% of the total variance. However, along PC2, which accounted for 23.04% of the total variance, XD-JK and XD-HY could be clearly separated from LQ-JK and LQ-HY. These results indicated that altitude has a significant influence on gene expression in peach fruit flesh.

Relative to LQ-JK and LQ-HY, XD-JK and XD-HY had 705 common DEGs and 1530 and 1102 unique DEGs, respectively (Figure 3B). KEGG pathway enrichment analysis of ‘Zhongtaojinkui’ revealed that these genes were predominantly enriched in 20 pathways, such as ‘plant hormone signal transduction’, ‘ascorbate and aldarate metabolism’, ‘alanine, aspartate and glutamate metabolism’, ‘cysteine and methionine metabolism’, and ‘starch and sucrose metabolism’, among others (Figure 3C). Similarly, ‘Zhongtaohongyu’ showed enrichment in pathways including ‘plant hormone signal transduction’, ‘fructose and mannose metabolism’, ‘pentose phosphate pathway’, ‘glutathione metabolism’, and ‘cysteine and methionine metabolism’, among others (Figure 3D). These results indicated that variations in titratable acid content in peach fruit from these two different altitudes could be attributed to differences in the expression levels of genes involved in organic acid metabolic pathways.

### 3.5. Expression Pattern of Key Genes Involved in Organic Acid Metabolism in Peach

**Figure 4:**
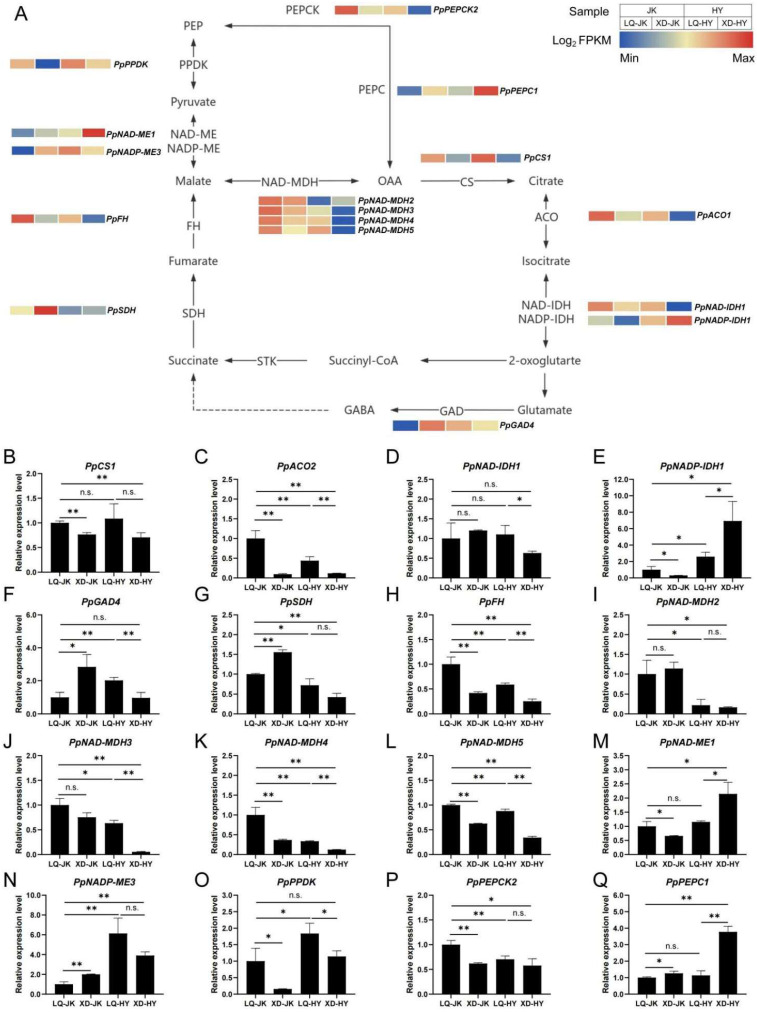
Expression pattern of key genes involved in organic acid metabolism in the fruits of Zhongtaojinkui’ and ‘Zhongtaohongyu’ from two peach-producing areas at different altitudes. (**A**) Metabolic pathway diagram for organic acids in peaches, integrated with transcriptome data. (**B**–**Q**) Results of real-time quantitative polymerase chain reaction (RT-qPCR) for 16 key genes involved in the metabolic pathways of organic acids in peaches. One or two asterisks indicate statistical significance according to Student’s *t*-test at the 0.05 or 0.01 level, respectively. “n.s.” stands for “not statistically significant”.

A total of 41 genes associated with the organic acid metabolism pathway were identified from the reference genome using BlastP (Figure 4A, Table S3). The synthesis and degradation of organic acids in fruit are dynamic processes. In this study, we further screened 16 key genes from transcriptome data based on the trend of total titratable acid content in four fruit samples. Subsequent fluorescence quantitative analysis results confirmed that the increase in organic acid content in peach fruit was related to the increase in the expression of *PpNAD-ME1*, *PpNADP-ME3,* and *PpPEPC1* in the synthesis pathway, and the decrease in the expression of *PpACO2*, *PpNAD-MDH2/3/4/5*, and *PpPEPCK2* in the degradation pathway (Figure 4B–Q). Notably, the expression levels of *PpACO2* and *PpNAD-MDH4/5* in the organic acid degradation pathway were significantly lower in peach fruit grown in high-altitude areas (XD-JK and XD-HY) than in those grown in low-altitude areas (LQ-JK and LQ-HY). These results indicated that the suppression of the organic acid degradation pathway and enhancement of the organic acid synthesis pathway may account for the higher organic acid content in peach fruit grown in high-altitude areas.

### 3.6. Potential Regulatory Networks and Models of Organic Acid Accumulation in Peach Fruit Grown in High-Altitude Areas

**Figure 5:**
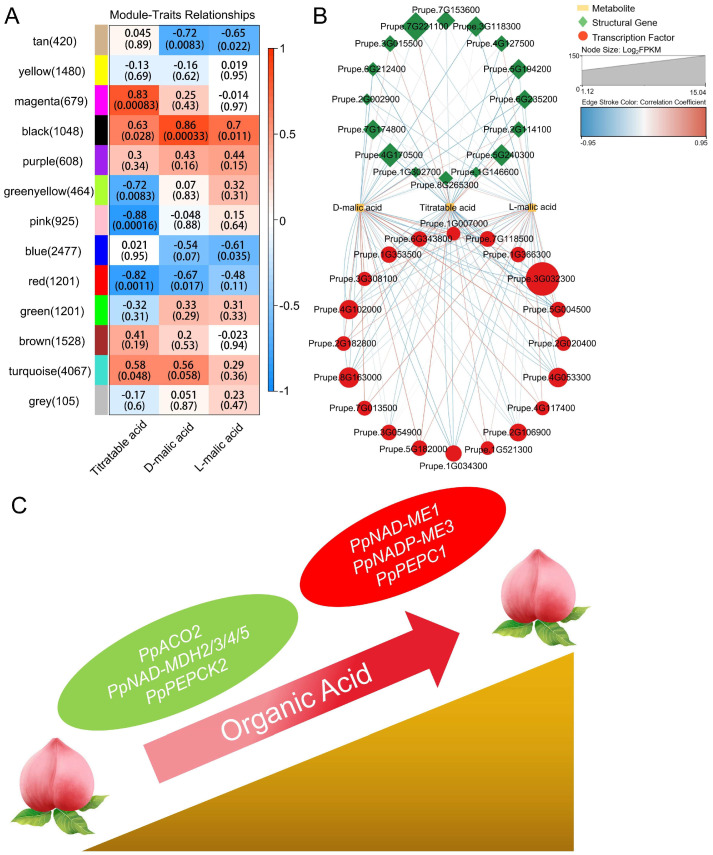
Based on weighted gene co-expression network analysis (**A**) and Pearson correlation analysis (**B**), key structural genes and transcription factors involved in the accumulation of organic acids in peach fruit were identified. A model for the significant accumulation of organic acids in peach fruit from high-altitude peach-producing areas is proposed (**C**): the down-regulation of key genes in the synthesis pathway and the up-regulation of key genes in the degradation pathway of organic acids ultimately lead to the accumulation of organic acids in the fruit.

Since the main titratable acid component accumulated in peach fruit is malic acid, and large amount of two isomeric forms of malic acid were detected in XD-HY (from a high-altitude production area), we identified the modules associated with titratable acid, D-malic acid, and L-malic acid through WGCNA (Figure 5A). Among these modules, the black and red modules exhibited the highest positive and negative correlations with these three organic acids, respectively. From each of the black and red modules, we selected ten transcription factors with the highest expression levels (Table S4). Subsequently, a correlation network was constructed by incorporating these 20 transcription factors, 16 structural genes involved in the organic acid metabolic pathway, and three organic acid traits (Figure 5B). Additionally, we proposed a model explaining the higher organic acid content in peach fruit grown at high altitudes (Figure 5C). The lower annual average temperature and stronger ultraviolet radiation in high-altitude areas were found to account for the increased expression of genes (*PpNAD-ME1*, *PpNADP-ME3*, and *PpPEPC1*) in the organic acid synthesis pathway and decreased expression of genes (*PpACO2*, *PpNAD-MDH2/3/4/5*, and *PpPEPCK2*) in the organic acid degradation pathway in peach fruit, ultimately resulting in the higher accumulation of organic acids.

## 4. Discussion

Organic acids are essential nutrients in peach fruit. Moderate sourness can provide a refreshing and appetizing sensation, whereas Asian people tend to prefer fruits with a pure sweet taste [18]. Hence, the content of organic acids in fruits has become a pivotal trait of breeding and cultivation in recent years. Peach originated from the relatively high-altitude western region of China, and the trait of organic acid content in the fruit has undergone a pronounced selection process by both natural and human selections during the domestication process [18]. As a result, the widely cultivated peach varieties in China exhibit consistently low levels of malic acid and citric acid accumulation [31]. Similarly, during the domestication of the Prunus genus fruit trees, such as apricots (*Prunus armeniaca* L.) [23], plums (*Prunus salicina* L.) [32], and Chinese cherries (*Prunus pseudocerasus* L.) [33], the organic acid content in the fruit has also undergone substantial domestication from high to low altitudes. In this study, the titratable acid content in the fruit of two peach varieties increased markedly after being introduced to the high-altitude peach-producing area, which is in agreement with the geographical distribution characteristics of peach germplasms with high organic acid contents during domestication [31]. Our findings, again, reinforce the notion that the natural environment has significantly reshaped the organic acid metabolome of peach fruit.

A variety of environmental factors can lead to an increase in organic acid content in plant fruits. Low temperatures may enhance the activity of organic acid metabolic enzymes in fruits and increase the storage capacity in vacuoles, thereby affecting the acidity of fruits [34]. Another study showed that low temperature induces the up-regulation of citric acid synthesis-related genes and the down-regulation of citric acid degradation-related genes in ponkan fruit (*Citrus reticulata* Blanco cv. Ponkan), resulting in a 1.4- to 1.9-fold increase in citrate content [35]. UV-C treatment can enhance the activity of glutamate decarboxylase (GAD) and up-regulate the expression of genes in the organic acid synthesis pathway in tomato (*Solanum lycopersicum* L.), thereby promoting the accumulation of organic acids and γ-aminobutyric acid (GABA) in fruits [36]. In this study, the low annual average temperature and intense ultraviolet light in the high-altitude mountainous areas of the Sichuan Basin are probably the primary reasons for the increase in organic acid content in peach fruit. Our findings may lay an important foundation for the future development of the peach industry and the selection of peach varieties for high-altitude peach-producing areas in southwest China.

Malic acid, quinic acid, and citric acid are the three most significant organic acids in peach fruit, and their proportions vary significantly among different varieties [7]. In this study, the predominant organic acid component in XD-HY with the highest organic acid content was still malic acid. Nevertheless, the organic acid profiles of the other three peach fruit samples exhibited notable differences from that of XD-HY, suggesting that there may be significant differences in malic acid metabolism in the flesh of peach grown at different altitudes. The transcriptome analysis revealed that the genes associated with the organic acid synthesis pathway were up-regulated, while those related to the organic acid degradation pathway were down-regulated in peach fruit from high-altitude peach-producing areas. Among them, *PpNAD-MDH3*/*4*/*5* were down-regulated in the same peach variety grown in high-altitude areas compared with that grown in low-altitude areas.

NAD-MDH catalyzes the reversible conversion between malic acid and OAA, which is crucial for the synthesis of malic acid [37]. The function of NAD-MDH varies significantly among different plant species [38]. During apple (*Malus pumila* Mill.) fruit development, the expression level of *MdMDH* is positively correlated with the activity of MDH, but not directly correlated with the malic acid content [37], suggesting that the malic acid metabolism in fruits may be influenced by other MdMDH family members. During the preservation of bananas (*Musa nana* Lour.) in the dark at low temperatures, the activity of the MDH enzyme increases gradually, but the content of malic acid decreases [39]. After silencing an *MDH* gene in arabidopsis (*Arabidopsis thaliana* L.), the malic acid content in the plant was not different from that of the wild type during the day, but was more than two times that of the wild type at night [40]. Similarly, the knockout of a cytosolic *NAD-MDH* gene in rice (*Oryza sativa* L.) resulted in a significantly higher malic acid content in the plant than that in the wild type at 9 days after flowering [41]. Therefore, it can be speculated that the expression of *NAD-MDH* decreases in response to environmental factors, such as relatively low temperatures in high-altitude areas, thereby weakening the degradation of malic acid in peach fruit. Since there is no stable genetic transformation system in peach, in vitro enzyme activity detection and transient transformation will help to further understand the function of *NAD-MDH* in peach fruit. In addition, we also constructed a potential regulatory network for organic acid metabolism in peach fruit based on WGCNA. Collectively, our findings offer new perspectives for advancing the study of peach fruit quality, particularly in high-altitude areas, and shed light on the potential regulatory network of organic acid metabolism in peach fruit.

## 5. Conclusions

During the introduction of some peach varieties from low-altitude to high-altitude peach-producing areas, the organic acid content in their fruits significantly increased. This study, employing targeted metabolomics of primary metabolites and transcriptomics, revealed a substantial accumulation of malic acid in the fruits of ‘Zhongtaohongyu’ peaches from high-altitude peach-producing areas, with the total organic acid content in these fruits being significantly higher than in other samples. Furthermore, the down-regulation of the key genes *PpNAD-MDH3/4/5* involved in malic acid metabolism may be the primary reason for the elevated malic acid accumulation in peach fruits from high-altitude peach-producing areas. This study elucidates the mechanism by which environmental factors enhance organic acid accumulation in peach fruits from high-altitude peach-producing areas from a multi-omics perspective and provides a theoretical basis for screening key genes related to organic acid metabolism in peach fruits.

## Data Availability

All primary metabolome data for this study are included in the Supplementary Materials. All RNA-seq data are deposited in the NGDC repository (https://ngdc.cncb.ac.cn/gsa, accessed on 20 September 2024) with the accession number CRA019041.

## Supplementary Materials

The following supporting information can be downloaded at https://www.mdpi.com/article/10.3390/plants13223171/s1: Table S1: Primers used for RT-qPCR analysis; Table S2: Identification of total primary metabolites in flesh of all peach samples; Table S3: A total of 41 genes associated with the organic acid metabolism pathway in peach samples; Table S4: The top ten transcription factors with the highest expression levels and their expression levels in the black and red.
